# Cost-benefit analysis of antibiofilm microbiological techniques for peri-prosthetic joint infection diagnosis

**DOI:** 10.1186/s12879-018-3050-8

**Published:** 2018-04-02

**Authors:** Carlo L. Romanò, Maria Teresa Trentinaglia, Elena De Vecchi, Nicola Logoluso, David A. George, Ilaria Morelli, Lorenzo Drago

**Affiliations:** 1 Centre for Reconstructive Surgery and Osteoarticular Infections, IRCCS Galeazzi Orthopaedic Institute, Milan, Italy; 2 Department of Environmental Science and Policy, University of Milan, Milan, Italy; 3 Laboratory of Clinical Chemistry and Microbiology, IRCCS Galeazzi Orthopaedic Institute, Milan, Italy; 4 Department of Trauma and Orthopaedics, University College London Hospitals, London, UK; 5 Department of Reconstructive Surgery of Osteo-Articular infections C.R.I.O. Unit, IRCCS Galeazzi Institute, University of Milan, Milan, Italy; 6 Laboratory of Clinical Chemistry and Microbiology, IRCCS Galeazzi Orthopaedic Institute and Laboratory of Clinical Microbiology, Department of Biochemical Sciences for Health, University of Milan, Milan, Italy

**Keywords:** Cost, Benefit, Economics, Diagnostic, Joint infection, Analysis, Biofilm

## Abstract

**Background:**

Implant-related infections, including those of peri-prosthetic joint (PJIs), osteosynthesis and other biomaterials, are biofilm-related. Pathogen identification is considered the diagnostic benchmark; however, the presence of bacterial biofilms makes pathogen detection with traditional microbiological techniques only partially effective. To improve microbiological diagnostic accuracy, some biofilm debonding techniques have been recently proposed. Aim of this health economics assessment study was to evaluate their economic impact on hospital costs.

**Methods:**

Direct and indirect hospital costs connected with the routine introduction of sonication and dithiothreitol treatment applied to hip and knee PJIs and of tissue cultures were examined. In particular the consequences of diagnostic inaccuracy, the opportunities, costs, and risks of each technique were calculated.

**Results:**

Considering an average of five samples per patient, processed separately with traditional tissue culture with or without sonication of prosthetic components, or pooled together using the MicroDTTect device (a close system for sample collection, transport and treatment with Dithiothreitol for microbial release from biofilm), the overall mean direct cost per patient was € 397 and € 393 for sonication or MicroDTTect, respectively, compared to € 308 for traditional tissue cultures. In terms of opportunity costs, MicroDTTect was the most effective technique, allowing for a 35% or 55% reduction in time required for sample treatment, compared to tissue cultures combined or not with sonication, respectively.

Pooling together direct and indirect costs associated with false positive and negative results of the different diagnostic techniques, unnecessary medical treatments and possible medical claims, MicroDTTect or sonication become increasingly cost-effective when the extra-costs, generated by diagnostic inaccuracy of traditional tissue culture, took place, respectively, in 2% or 20% or more of the patients.

**Conclusions:**

This is the first study specifically focused on the economic impact of the routine clinical use of microbiological antibiofilm sampling and processing techniques in orthopaedics. Although our results may suffer from a potential country and hospital bias, as the data collection process for direct and indirect costs is specific to each institution and country, this analysis highlights the potential economic advantage to hospitals associated with the routine introduction of antibiofilm techniques for microbiological diagnosis of PJI.

## Background

Implant-related infections, including those affecting joints (PJI), osteosynthesis and other biomaterials, are biofilm-related [1–3].

Intra-operative clinical diagnosis and pathogen identification is considered the diagnostic benchmark [4, 5], however the presence of bacterial biofilm makes pathogen detection with traditional microbiological techniques only partially effective with high rates of FP and FN. In order to improve the accuracy of microbiological assays, methods for detachment of bacteria from biofilm formed on prosthetic implants have been developed in the last decade, including sonication [6–11], and, more recently, chemical debonding technique using D,L dithiothreitol (DTT) [12–16].

Comparison of DTT treatment with sonication has evidenced a similar rate in FP (5.88%) and a higher frequency of FN with sonication (28.6%) than with DTT (14.3%) [13]. Superiority of sonication in comparison with tissue culture has been reported by many authors, who generally underline the lower sensitivity of tissue samples (ranging from 61% to 76%) with respect to sonicated implants (77–95%) [6, 8, 17–19]. Moreover, FP increase when culture of tissue samples is performed (23.5%), probably due to the higher risk of contamination associated with tissue sampling [13]. Health technology assessments are increasingly used to inform coverage, access, and utilization of medical technologies [20], as for example molecular diagnostics [21] and medical devices [22]. While both preclinical and clinical testing showed the ability of these technologies to improve diagnostic accuracy in implant-related infections, compared to current tissue sampling, little is known about the economic impact of their introduction in routine diagnosis on hospital costs.

To the best of our knowledge, only two contributions evaluate the economic impact of different diagnostic techniques for PJIs. Diaz Ledezma et al. [23] developed a multicriteria analysis to assess the costs, opportunities and benefits of three different diagnostic strategies to be implemented in an ambulatory setting. Then, Peel et al. [24] developed a decision tree model to evaluate the impact, in terms of laboratory process time and costs, of performing periprosthetic tissues culture in blood culture bottles instead of using conventional techniques. Still, none of these contributions has addressed the economic impact of antibiofilm microbiological techniques in orthopaedics and trauma.

Hence, this analysis aimed to fill this gap by assessing the total economic impact of introducing antibiofilm microbiological techniques to diagnose hip and knee PJIs, comparing their direct and indirect hospital costs with the current economic standard offered by routine microbiology testing. In our setting, the main source of additional costs is precisely a wrong diagnosis, thus ruling out all other possible complications that may emerge in the treatment of PJIs (infection persistence or malpractice, among other factors).

## Methods

The decision-analytic modelling approach used to conduct the cost-effectiveness analysis presented here was based upon the framework of Diaz-Ledezma et al. [23], who assessed the effectiveness of three different diagnostic strategies to detect PJI in an ambulatory setting. Their framework relies on an Analytic Hierarchy Process supporting a multicriteria decision. They evaluated the benefits, opportunities, economics costs, and risks of each strategy, and assigned to each criterion a specific priority, or weight, in order to identify the best diagnostic approach.

Our analysis focused instead on three different intra-operative diagnostic approaches. We compared, in fact, the economic impact of tissue culture with that of two antibiofilm technique: sonication, and DTT, using MicroDTTect® system (4i Srl, Monza, Italy), a close system for intra-operative tissue and implant sampling, transport and antibiofilm processing.

For each technique, we evaluated the consequences of diagnostic inaccuracy, relatively to routine PJI microbiological diagnosis. In a similar vein to Diaz-Ledezma et al. [23], we defined and evaluated the opportunities, costs, and risks of each technique. Still, we deemed these criteria all equally important, and no specific weight or priority was assigned: it follows that the diagnostic performance was evaluated considering jointly all these three different elements, and the resulting best diagnostic was optimal with respect to all the three factors considered.

The opportunities of each technique reflected the opportunity cost of each technique, as expressed by the Laboratory time-opportunity cost (‘turn-around time’, L-TAT), which is the time needed by biologists and technicians to perform the diagnostic activity.

With regards to the cost assessment, we adopted a broader definition which distinguished between direct and indirect costs. The former cost voice included both the material and other operational costs, whereas the latter referred to the additional costs (medical and legal) that might stem from a wrong diagnosis. Moreover, we distinguished between the indirect estimated medical costs resulting from diagnostic inaccuracy (i.e., FN – FP induced medical and surgical treatments, hospital stay, infection recurrence/ persistence) and the indirect legal costs, that accounted for the incidence and costs related to medico legal claims following a wrong diagnosis. The resulting medical and legal costs were weighted by the probability of having a wrong diagnosis. Hence, our definition of risk had a double interpretation: if, on one side, it reflected the probability of a wrong diagnosis, just as in Diaz Ledezma et al. [23], on the other side it translated this risk into an economic one, i.e. the economic disbursement that hospital might face for each wrong diagnosis.

**Fig. 1:**
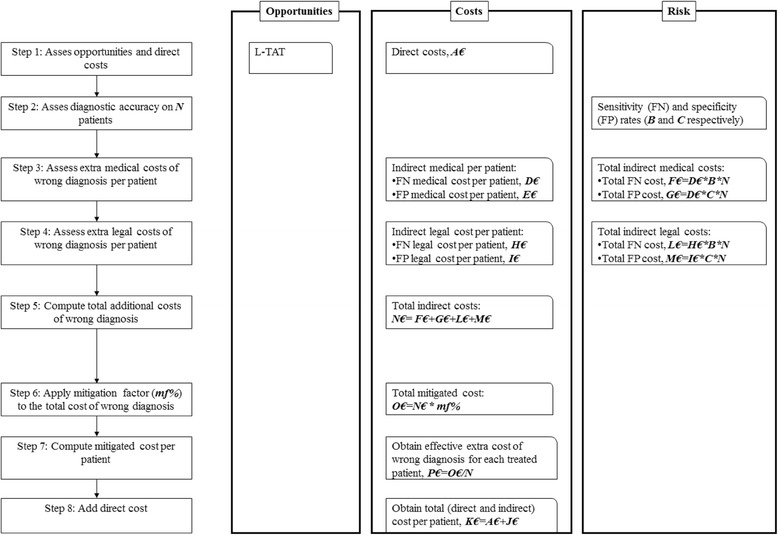
Decision tree model and steps undertaken to assess the economic impact of each alternative diagnostic technique

PJIs were defined according to the criteria established by the International Consensus Meeting of Philadelphia: presence of a sinus tract communicating with the prosthesis, isolation of the same microorganisms from at least 2 samples or fulfilment of three of the following requisites: acute inflammation evidenced by histology of periprosthetic tissues, one positive culture, high synovial leukocyte count or a ++ result on the leukocyte esterase strip, high percentage of synovial neutrophils, elevated erythrocyte sedimentation rate (ESR) and C-reactive protein (CRP) [25]. All the steps adopted to perform our analysis are displayed in Fig. 1 and discussed in details below.

### Direct costs and opportunities

As displayed in Fig. 1, we initially assessed the opportunities, in terms of L-TAT, and direct costs for each diagnostic strategy (step 1). To this purpose, for each patient we considered 5 tissue samples plus 1 prosthetic implant undergoing sonication, processed separately or pooled together as occurs using the MicroDTTect device. Sonication may in fact only be applied to implants but is not suitable for organic tissues, while MicroDTTect with chemical biofilm debonding allows to free pathogens from both tissues and implants that, therefore, can then be processed together [13, 14, 16].

All direct costs were collected at the Galeazzi Orthopaedic Institute in Milan, Italy, on a case study of 20 patients, for a total of 100 tissue samples and 20 implants. These voice costs cover costs for materials (plastics and culture media) and laboratory staff activities (determined according to the salary of microbiologists and technicians in our Institute). To this aim, we reviewed the impact of the L-TAT in our microbiology laboratory, which was defined as the time taken from sample arrival to the laboratory to the availability of a report to the clinician. This was calculated by prospectively documenting the dates and times when the sample was received in the laboratory, the report signed by the microbiologist, the report sorted by the laboratory clerical staff, and the final report received by the clinician [26]. Moreover, for each technique, we also calculated the time required by laboratory personnel to carry out all the operations, from the pre-analytical phase and sampling preparation to the final control and validation of the result.

### Indirect costs: estimated medical costs of diagnostic inaccuracy

The additional medical costs that arise from microbiological diagnostic inaccuracy were considered as indirect costs and were extracted from the most recent literature. In fact, failure to identify the pathogen in a PJI (FN) may result in no or inadequate antibiotic treatment and higher risk of infection recurrence/persistence. Samples contamination and false pathogen identification (FP) may be associated with unnecessary antibiotic therapy and post-surgical surveillance and monitoring. Additionally, if the prosthesis was infected but the result was a wrong isolate, i.e. the isolate was not the real pathogen but a contaminant, the patient may have received inadequate or ineffective antibiotic treatment, with possible treatment failure and infection recurrence or persistence. In extreme circumstances, the patient may require further surgery to treat the misdiagnosed PJI.

Before quantifying precisely these additional medical costs, we first had to assess the diagnostic accuracy of each technique, as prescribed by the algorithm displayed in Fig. 1 (step 2). Hence, we assessed the risk of having a wrong diagnosis in terms of either FN or FP results. To this purpose, we considered the relative sensitivity and specificity of the diagnostic techniques as reported in a previous study comparing sonication with DTT treatment.

We then proceeded with the assessment of the indirect additional medical costs that each wrong diagnosis generates (step 3 in Fig. 1). As for FN results, these voice costs include the prolonged antibiotic treatment and the surgical costs implied by infection recurrence. The indirect medical cost stemming from a FP diagnosis accounts for the prolonged antibiotic treatment as well as for the post-surgical surveillance and monitoring. These different cost specifications were extracted from the most recent literature [23, 27, 28].

To translate these indirect costs into a measure of economic risk, we multiplied the medical cost of each FN or FP case by the corresponding total potential incidence of FN and FP rates on a given patient cohort of size N (step 3 in Fig. 1). Then, we proceeded with the assessment of medico-legal costs of diagnostic inaccuracy (step 4 in Fig. 1). To the best of our knowledge, there are no dataset that specifically assess the medico-legal claims following a wrong diagnosis after a hip or knee replacement. To overcome this issue we exploited the data on the medico-legal claims for post-surgical infection following a wrong diagnosis after a hip or knee surgery in our Institute, from 2010 to 2013. To estimate the potential impact on medico-legal cost reduction, that may result from a more accurate microbiological technique, we first retrieved the frequency and economic magnitude of the claims occurred between 2010 and 2013. Based on these data, we were then able to estimate the potential economical medico-legal cost of PJI inaccurate diagnosis. Following the approach adopted in step 3, we quantified the total economic risk of legal claims by multiplying the unitary legal claim of a FN or FP patient by the total potential number of FN and FP in the patient cohort of size N.

As a last step, we computed the total indirect cost, which accounts for both the additional medical and legal cost that may potentially stem from a wrong diagnosis (step 5 in Fig. 1).

### Algorithm to calculate the economic impact of microbiological techniques

As stressed above, Fig. 1 displays the algorithm used to calculate the overall economic impact of microbiological diagnostic accuracy; direct and indirect costs, as previously defined, were included and weighted on the basis of the sensitivity and specificity of the three diagnostic techniques under study (sonication, MicroDTTect® and tissue culture).

According to the proposed algorithm, the overall estimated FN and FP induced costs for 100 patients, undergoing hip or knee revision surgery, were respectively calculated, according to the following formulas (step 3 in Fig. 1):

$$ Estimated\  FN\  Medical\ Costs\ x\ \left( Number\ of\ treated\ patients\ x\  FN/100\right) $$

$$ Estimated\  FP\  Medical\ Costs\ x\ \left( Number\ of\ treated\ patients\ x\  FP/100\right) $$

Then, for each patient, the potential medico-legal cost has been calculated according to the following formula (step 4 in Fig. 1):

$$ Total\ amount\ paid\ for\ medico- legal\ claims\ for\ post- surgical\ infections\ after\  hip\  or\ knee\ revision\ surgery\ from\ 2010\  to\ 2013\ x\  FN\  or\  FP\  rate/ Number\ of\ aseptic\  hip\  or\ knee\ revision\ performed\ from\ year\ 2010\  to\ 2013. $$

Since not all wrong diagnosis will actually generate additional and indirect costs, we applied to the total indirect cost of a wrong diagnosis a “*mitigation factors*”, corresponding to the percentage of patients in which the diagnostic inaccuracy will effectively produce an extra-cost (step 6 in Fig. 1). In fact, for example, an intra-operative FN may not have any clinical impact in a given patient and hence no further medical costs, if the infection could be effectively diagnosed by other means (e.g.: frozen sections or intra-operative synovial fluid examination). Similarly, not all misdiagnosed pre-existing infection will produce medico-legal claims. The occurrence of these variables may then “mitigate” the impact of the microbiological inaccuracy of a given diagnostic technique; this is mathematically expressed by the *mitigation factor*, that may range from 0% (diagnostic inaccuracy has no economic impact on any treated patient) to 100% (diagnostic inaccuracy has a full economic impact on all treated patients). In this analysis, we considered four different mitigation factors (1%, 2%, 10%, and 20%) to account for an increasingly share of wrong diagnosis actually generating additional costs.

The mitigation factor was then applied to the overall cost of intra-operative diagnostic inaccuracy for the treated patients, as derived in step 5. The resulting mitigated cost was then given by the following equation (step 6 in Fig. 1):

$$ \left( Overall\ estimated\  FN\  induced\ costs+ Overall\ estimated\  FP\  induced\ costs+ Overall\ estimated\ medico- legal\ costs\right)\ast Mitigation\ factor $$

We then divided this cost of intra-operative diagnostic inaccuracy by the total number of treated patient (step 7 in Fig. 1), to retrieve the effective cost of diagnostic inaccuracy for the single patient, which was defined as:

$$ Cost\ of\ intra- operative\ diagnostic\ inaccuracy/ Number\ of\ treated\ patients $$

Finally, the total direct and indirect costs per patient will correspond to the sum of the estimated cost of intra-operative diagnostic inaccuracy per patient and the cost of microbiological analysis per patient (step 8 in Fig. 1).

## Results

### Opportunity

In terms of opportunities, or L-TAT, MicroDTTect® was the most effective diagnostic strategy as the simultaneous collection and processing of multiple samples was less time consuming and allowed for a significant abatement of operational times (58 min vs. 131 min of sonication and 90 min of tissue culture). On aggregate terms, MicroDTTect led to a 35% decrease of the time required for processing and analysing samples, relatively to tissue culture, or 55% if compared to sonication.

### Direct costs

The mean calculated direct costs of standard tissue culture, including all consumables and L-TAT, was € 61.5 per processed sample or € 307.5 per patient (5 processed samples) [29, 30]. When using antibiofilm techniques, measured direct costs of microbiological analysis raised to € 89.6 per sample, or € 397.1 per 5 samples plus sonication of the retrieved implant, or € 393.3 per patient when using MicroDTTect and pooled sampling (Table 1).

**Table 1:** Direct laboratory costs at our Institution for standard tissue culture, sonication and MicroDTTect

Direct costs (*N* = 20 treated patients)/sample			Tissue Culture (5 samples)		Tissue Culture (5 samples) + Sonication		MicroDTTect (pooled 5 samples)	
Materials	€/hour	Activities / Product	Minutes	€	Minutes	€	Minutes	€
		Container		1		2		350
		Tubes		1		1		
		Plates and broths		5		5		5
		Card ID and antibiogram		10		10		10
		Loops, pipettes and others		2		2		
		Sonicator depreciation and maintenance				10		
		Total		19		30		365
Technician	25.0	Pre-analytical phase	4	1.7	4	1.7	2	0.8
		Sample preparation/processing	27	11.3	10	4.2		
		Sonication			18	7.5		
		Centrifugation	15	6.3	30	12.5	15	6.3
		Seeding	5	2.1	30	12.5	5	2.1
		Microscope slide preparation	10	4.2	10	4.2	10	4.2
		Vitek loading	10	4.2	10	4.2	10	4.2
		Total	71	29.7	112	46.8	42	17.6
Biologist	40.0	Pre-analytical phase	4	2.8	4	2.8	1	0.7
		Plates reading	3	2	3	2	3	2
		Broth control for 15 days	10	6.7	10	6.7	10	6.7
		Control and final validation	2	1.3	2	1.3	2	1.3
		Total	19	12.8	19	12.8	16	10.7
Total per Sample			90	61.5	131	89.6	58	393.3
Total per Patient (5 Samples)				307.5		397.1		393.3

### Risk

Diagnostic accuracy of the techniques under study was evaluated according to Drago et al. [13] and summarized in Table 2. More specifically, both DTT and sonication were associated with a 5.9% FP incidence, compared to the 23.5% of tissue culture, while the FN rate was equal to 14.3% when using DTT, compared to 28.6% when using either sonication or tissue cultures. These data are in line with those published by other authors [14, 31, 32].

**Table 2:** Diagnostic accuracy of MicroDTTect and sonication compared to tissue culture, according to [13]

	Sensitivity	Specificity	Positive predictive value	Negative predictive value	Accuracy
MicroDTTect	85.7%	94.1%	94.7%	84.2%	89.5%
Sonication	71.4%	94.1%	93.7%	72.7%	81.6%
Tissue culture	71.4%	76.5%	78.9%	68.4%	73.7%

### Indirect medical costs

Failure to identify the responsible for a PJI or a FN result have the following potential clinical consequences: possible inadequate antibiotic treatment and risk of infection recurrence/persistence. The costs resulting from inadequate and often prolonged antibiotic treatment were estimated on average as € 4500 (range € 2000 to € 8000), according to Hernández-Vaquero (2013) [28], while the potential costs of additional surgical and medical treatments, as well as a potential prolonged hospital stay, associated with infection recurrence were on average approximately € 45,000 (range 40,542 to 52,555) [23, 27, 28]. The overall estimated indirect cost of a FN result was then set at € 49,500.

Sample contamination and FP results may also lead to prolonged antibiotic treatment; moreover, unnecessary post-surgical surveillance and monitoring was associated to average costs of € 4000 (range € 3000 to € 5000) [23, 27, 28], so the overall estimated indirect cost of a FP result was set at € 8500.

### Indirect legal costs

Concerning medico-legal costs arising in the period considered (2010–2013), 1083 hip or knee revision surgeries were performed at our Institute; of these, 513 were considered as aseptic and 570 septic. During the same years, a total of 13 medico-legal claims following a hip (*N* = 3) or knee (*N* = 10) arthroplasty revision were settled at our Institute, due to post-surgical infections; the total amount paid over these three years was € 1,192,456 or € 298,114/year, with an average amount of € 91,727 paid to the recipient. Since 28% of these post-surgical infections were misdiagnosed PJIs, due to intra-operative FN results, the estimated medico-legal cost of diagnostic inaccuracy was € 333,887/year or € 650.85/patient (€ 1,192,456 × 28% / 513). A reduction of FN results from 28% to 14%, as determined by the introduction of the MicroDTTect, would potentially result in a medico-legal cost per patient of € 325.42.

To estimate the unitary legal cost of a FP result, we divided the total amount of liquidated claims by the number of septic revisions, and then multiplied this number by the rate of FP results. When tissue culture was used as a diagnostic strategy, the additional legal cost was €418.17/patient (€ 1,192,456 × 23% / 570). Sonication and MicroDTTect, by reducing the incidence of FP results from 23% to 6%, could abate the legal cost per patient to €125.52.

We then weighted the medical and legal costs likely arising from a wrong diagnosis by the effective incidence of FN and FP results (respectively steps 3 and 4 in Fig. 1). These total measures of medical and legal economic risk were then aggregated to compute the total cost of wrong diagnosis (step 5 in Fig. 1).

### Total indirect costs

As reported in Table 3, for every 100 patients treated each year, the total cost of a wrong diagnosis with a tissue culture would amount to €1,6 mln; with sonication and MicroDTTect the total potential costs would be reduced to respectively €1,4 mln and €0,7 mln.

**Table 3:** Comparative direct and indirect costs of traditional tissue cultures, sonication and MicroDTTect

Step	Assessment		Tissue culture	Sonication	MicroDTT
0		Number of treated patient	100	100	100
1	Opportunities	LTAT (per patient)	90	131	58
1	Costs	Direct costs per patient	308	397	393
2	Risk	FN %	28%	28%	14%
2	Risk	FP %	23%	6%	6%
3	Costs	Indirect medical cost per FN patient	€ 49,500.00	€ 49,500.00	€ 49,500.00
3	Costs	Indirect medical cost per FP patient	€ 8500.00	€ 8500.00	€ 8500.00
3	Economic risk	Total indirect medical cost per FN	€ 1,386,000.00	€ 1,386,000.00	€ 693,000.00
3	Economic risk	Total indirect medical cost per FP	€ 195,500.00	€ 51,000.00	€ 51,000.00
4	Costs	Indirect legal cost per FN patient	€ 650.85	€ 650.85	€ 325.43
4	Costs	Indirect legal cost per FP patient	€ 481.17	€ 125.52	€ 125.52
4	Economic risk	Total legal cost (both FN and FP)	€ 18,223.89	€ 18,223.89	€ 4555.97
4	Economic risk	Total legal cost per FP patient	€ 11,066.83	€ 753.13	€ 753.13
5	Costs	Total cost of wrong diagnosis	€ 1610,790.72	€ 1455,977.02	€ 749,309.10

Still, not all FN and FP actually generate additional costs. To better estimate the real and effective economic incidence of wrong diagnosis on hospital budgets, we apply four different mitigation factors, ranging from 1% to 20%.

### Mitigation factor and actual incidence of wrong diagnosis

According to these simulations, MicroDTTect appears to be in economic balance, compared to tissue cultures, already with a 1% mitigation factor. In this case, total costs with MicroDTTect and tissue culture respectively amounted to €467.93 and €468.58. MicroDTTect started making economic savings for the hospital from a *mitigation factors* of 2%.

Table 4 shows that sonication reached its economic balance starting with a *mitigation factor* of 10%. In this case, the total direct and indirect costs with sonication would amount to €1852 per patient, 3% less than the costs implied by tissue culture.

**Table 4:** Comparative direct and indirect costs of traditional tissue cultures, sonication and MicroDTTect with four different mitigation factors

	Tissue culture	Sonication	MicroDTT
Total cost of wrong diagnosis	€ 1610,790.72	€ 1455,977.02	€ 749,309.10
Mitigation factor	1%		
Total effective cost of wrong diagnosis (100 patients)	€ 16,107.91	€ 14,559.77	€ 7493.09
Effective cost of wrong diagnosis per patient	€ 161.08	€ 145.60	€ 74.93
Direct cost	€ 307.50	€ 397.00	€ 393.00
Total effective cost per patient	€ 468.58	€ 542.60	€ 467.93
Mitigation factor	2%		
Total effective cost of wrong diagnosis (100 patients)	€ 32,215.81	€ 29,119.54	€ 14,986.18
Effective cost of wrong diagnosis per patient	€ 322.16	€ 291.20	€ 149.86
Direct cost	€ 307.50	€ 397.00	€ 393.00
Total effective cost per patient	€ 629.66	€ 688.20	€ 542.86
Mitigation factor	10%		
Total effective cost of wrong diagnosis (100 patients)	€ 161,079.07	€ 145,597.70	€ 74,930.91
Effective cost of wrong diagnosis per patient	€ 1610.79	€ 1455.98	€ 749.31
Direct cost	€ 307.50	€ 397.00	€ 393.00
Total effective cost per patient	€ 1918.29	€ 1852.98	€ 1142.31
Mitigation factor	20%		
Total effective cost of wrong diagnosis (100 patients)	€ 322,158.14	€ 291,195.40	€ 149,861.82
Effective cost of wrong diagnosis per patient	€ 3221.58	€ 2911.95	€ 1498.62
Direct cost	€ 307.50	€ 397.00	€ 393.00
Total effective cost per patient	€ 3529.08	€ 3308.95	€ 1891.62

## Discussion

To our knowledge, this is the first study specifically focused on the potential economic impact of the routine clinical use of microbiological antibiofilm sampling and processing techniques in orthopaedics.

Health technology assessment is considered among the main priorities within the European Community, allowing to better allocate the resources and to drive healthcare policies in a more scientifically and transparent way, but economic analysis concerning microbiological techniques and technologies are lacking [22].

The methodology used to calculate direct costs is in line with the most recent recommendations of European guidelines concerning health technology assessment [33, 34], as well as with other previous studies [23, 24]. Our cost-assessment relied on the identification of the four different criteria also assessed by [23]: opportunities, risks, costs, and economic risks. Differently from [23], who performed a multicriteria analysis assigning different weights and priorities to the above-mentioned criteria, our final assessment equally weighted all these different elements, and provided a final assessment which was not affected by changes in the priorities assigned.

Our only focus was in fact on the economic impact of alternative diagnostic strategies: as such, our analysis was a simplified cost-benefit analysis, in which the unique pay-off was an economic one, ruling out, for the time being, any effect on patients’ life quality and utility. The results of our study point out, for the first time, how the introduction in the clinical setting of antibiofilm technologies specifically designed to diagnose PJIs can be cost-effective, when considering direct and indirect costs, whenever indirect costs have an impact on only 1% or 10% of the patients, respectively for MicroDTTect or sonication. Moreover, chemical debonding, by pooling together tissue samples and implants, is proved to be also time-effective.

The results of Diaz-Ledezma et al. [23] are instead sensible to the priorities and weights assigned to each criterion: if opportunities, benefits and risks are more relevant than the economic impact, then clinicians should proceed with the screening with serum markers followed by arthrocentesis in positive cases; if instead the economic aspect is the most relevant, than an immediate arthrocentesis resulted as the best alternative.

Our analysis is in line with the results obtained by [24], whose decision tree model showed that new technologies improving the diagnostic sensitivity and rapidity of detection can reduce the laboratory process time and generate significant economic savings.

Despite the robustness and validity of our results, the following limitations of the present study should be taken into consideration.

First of all, we acknowledge that our results may suffer from a country and hospital bias. In fact, the direct and indirect costs (both medical and legal) used for our calculations are hospital and country specific. In fact, hourly labour cost and direct costs of materials may differ from one country to the other or among various institutions [35, 36]. Moreover, microbiological techniques are not standardized across different centres and a great variability concerning the diagnostic procedures and accuracy of PJI has been previously reported [37]. As an example, direct costs were calculated in our study assuming an average of 5 peri-prosthetic tissue samples. This is in accordance with the widely accepted methodology, originally described by Atkins and co-workers [29] and part of the official IDSA and ICM guidelines [25, 30]. However, the ideal number of samples is still under debate and some authors have recently suggested that four or even three samples can be sufficient if processed using an automated blood culture bottle system [38].

With regards to the direct costs and opportunities collection process, we acknowledge the limited sample size (20 patients). It is worth stressing that the actual sample size is greater than 20, as it consists of 5 tissues samples and 1 prosthetic impact, for a total of 100 + 20 observations. Furthermore, we would recall that this is a pilot study, whose methodological framework can be rapidly and easily extended to a larger sample.

Also, the indirect costs’ calculation can be prone to bias and errors. In particular, we have no data concerning the real-life impact of misdiagnosis and related costs. Hence, in the present study, to calculate the relative economic burden of wrong treatments, originating from inaccurate diagnosis, several assumptions have been made, based on previous papers and reports; these assumptions may not be completely accurate and reproducible for every institution and for every country.

Similarly, concerning the quantification of the medico-legal risk, this has been calculated on the basis of our Institute database, which may not be assumed as necessarily representative of other hospitals. Furthermore, while wrong diagnosis and infection are a well-known potential trigger of medico-legal claims [39–41], the frequency of medico-legal claims is highly variable in different countries. Moreover, we are aware that our definition of indirect costs is limited to the short-term additional costs stemming from a wrong diagnosis. As such, our analysis does not account for other potential sources of costs that may arise over a longer temporal horizon, such as malpractice, infection recurrence and prolonged hospitalization. Despite being relevant in everyday hospital practice, these other factors have been excluded from our analysis, which rather quantifies the short-term economic consequences of wrong diagnosis of different diagnostic strategies. The model we propose here provides a static approach, which is not compatible with a long-term perspective, which would require instead a dynamic Markov utility model.

## Conclusions

This cost analysis highlights the potential benefits of antibiofilm technologies that may offer a more accurate pathogen identification, leading to an improvement in the management of implant-related infections with a substantial economic balance or advantage.

## Abbreviations

DTT: D,L Dithiothreitol
FN: False negative result
FP: False positive result
IDSA: Infectious Diseases Society of America
L-TAT: Laboratory turn around time
PJI: Prosthetic joint infection
